# Radiation Resilient Synthetic Antiferromagnets‐Based Neuromorphic Device for Sea Surface Temperature Reconstruction

**DOI:** 10.1002/advs.76388

**Published:** 2026-06-27

**Authors:** Mingxu Song, Jiahao Liu, Ruisheng Hu, Teng Xu, Aihua Tang, Zhihong Zhu

**Affiliations:** ^1^ College of Advanced Interdisciplinary Studies National University of Defense Technology Changsha China; ^2^ College of Meteorology and Oceanography National University of Defense Technology Changsha China; ^3^ Anhui Province Key Laboratory of Low‐Energy Quantum Materials and Devices High Magnetic Field Laboratory Hefei Institutes of Physical Science Chinese Academy of Sciences Hefei China; ^4^ Nanhu Laser Laboratory National University of Defense Technology Changsha China

**Keywords:** neuromorphic computing, radiation resistance, sea surface temperature reconstruction, spin orbit torque, synthetic antiferromagnet

## Abstract

Reconstruction of sea surface temperature is critical for marine monitoring, yet conventional edge devices based on complementary metal–oxide–semiconductor (CMOS) technology suffer from memory‐wall bottlenecks and radiation vulnerability in harsh marine environments. Here, we propose a neuromorphic computing framework based on radiation‐tolerant synthetic antiferromagnetic (SAF) synaptic devices through physical–algorithmic co‐design to achieve robust sea surface temperature reconstruction. The fabricated Ta/Ir/Fe_0.65_Tb_0.35_/Ru/Co/Pt/Ta‐based SAF devices enable field‐free magnetization switching via spin–orbit torque, exhibiting multilevel conductance states that naturally emulate synaptic and neuronal functions. Notably, these devices retain over 92% of their performance after 1 Mrad (Si) γ‐irradiation, demonstrating inherent radiation tolerance arising from strong antiferromagnetic exchange coupling. By mapping the nonlinear conductance response of SAF onto the cross‐attention mechanism of a Perceiver IO architecture, we achieve accurate reconstruction of sea surface temperature fields from sparse sensor inputs. On the National Oceanic and Atmospheric Administration dataset, our system attains a root‐mean‐square error below 2°C—competitive with deep learning baselines—while projections indicate a potential reduction in energy consumption by an order of magnitude. This work not only advances the application of neuromorphic computing in marine science, but also provides a promising pathway toward “environmentally adaptive intelligent computing”.

## Graphical abstract

By leveraging strong antiferromagnetic coupling, radiation‐tolerant synthetic antiferromagnetic synaptic devices are developed. Their intrinsic nonlinearity emulates neuronal activation, while linear and symmetric conductance modulation mimics synaptic plasticity. A physics–algorithm co‐design enables accurate sea surface temperature reconstruction, providing a promising pathway toward “environmentally adaptive intelligent computing”.

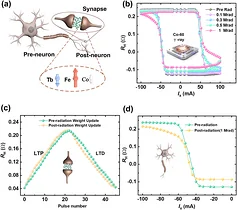

## 1 Introduction

Accurate reconstruction of sea surface temperature (SST) not only aids in understanding global energy balance and ocean circulation dynamics, but also provides critical data support for detecting El Niño‐Southern Oscillation phenomena and forecasting typhoon paths [1]. Currently, addressing this complex inversion problem primarily relies on two technical approaches including computational platforms based on metal–oxide–semiconductor (CMOS) technology on the hardware and increasingly sophisticated neural network algorithms on the software. However, these solutions both face key bottlenecks: traditional CMOS suffers from memory‐wall‐induced high data access energy (failing long‐term low‐power demands) and radiation‐triggered soft errors (disrupting reconstruction); GPUs/FPGAs, while accelerating algorithms, consume watt‐level power, incompatible with passive low‐power marine devices like buoys.

Neuromorphic spintronic devices utilize information stored in the magnetization vector of a magnetically ordered medium instead of charge [2, 3], endowing them with intrinsic radiation tolerance to ionizing radiation [4, 5, 6], a key advantage for marine and near‐space environments [7, 8, 9]. Meanwhile, their inherent physical properties closely mimic the dynamic behaviors of biological synapses and neurons, making them a promising alternative for low‐power edge neuromorphic computing in the post‐Moore's Law era [10, 11]. In recent years, artificial synaptic and neuronal devices have been realized by using spintronic devices, including magnetic tunnel junctions [12, 13], domain wall (DW) devices [14, 15, 16] and magnetic skyrmions [17, 18, 19]. In particular, SAF‐based neuromorphic devices manipulated by spin–orbit torque (SOT) stand out for their scenario‐adaptive merits: structurally, SAF comprises two antiferromagnetically coupled ferromagnetic (FM) layers with near‐zero net magnetic moment, rendering it strongly immune to external magnetic field interference (ideal for the ocean's complex geomagnetic environment) and enhancing thermal stability [20]; meanwhile, SOT enables nanosecond‐scale [21], energy‐efficient magnetization switching—laying the physical foundation for rapid, low‐power “neuron” spiking and “synaptic” weight modulation [21, 22, 23]. Furthermore, the dynamic properties of antiferromagnetic order endow ultrafast response capabilities and theoretically superior radiation tolerance [24, 25, 26], making SOT‐driven SAF devices prime candidates for high‐robustness, ultra‐low‐power neuromorphic computing. To date, most research has focused on characterizing individual device electrical properties [27, 28, 29, 30], demonstrating basic logic functions [11, 31, 32, 33], validating proof‐of‐concept in simple pattern recognition (e.g., MNIST) [34, 35, 36], and investigating ion irradiation/displacement damage effects on devices [37, 38]. However, integrating their unique physical merits—especially environmental robustness and high energy efficiency—with advanced neural networks to tackle complex spatiotemporal inversion tasks of practical marine significance remains largely unexplored.

In the work, we develop radiation‐resilient Ir/FeTb/Ru/Co/Pt SAF synaptic and neuronal devices via physical‐algorithmic co‐design, tailored for SST reconstruction in harsh marine environments. Through utilizing the anomalous Hall effect (AHE) and vibrating sample magnetometer (VSM) measurements, we experimentally demonstrate the implementation of a class of full compensated SAF with perpendicular magnetic anisotropy (PMA) at room temperature, alongside current‐induced SOT‐driven field‐free magnetization switching. In particular, testing magnetic moment switching behavior under varying radiation doses confirms the outstanding radiation resilience of the devices. Furthermore, by tuning the amplitude/duration of write current pulses, we mimic and modulate the neuromorphic plasticity of the SAF devices: when acting as a synapse, it exhibits a nearly linear, symmetric weight update curve; when functioning as a neuron, it displays a nonlinear sigmoid activation function. Crucially, by integrating these advantages, we establish a connection between the nonlinear response characteristics of the SAF devices and the cross‐attention mechanism of the Perceiver IO architecture, and perform simulations for SST field reconstruction. This approach not only validates the feasibility of SAF devices for complex computational tasks but also explores a path for synergistic optimization between algorithmic requirements and device characteristics.

## 2 Results and Discussion

### 2.1 Preparation and Characterization of SAF Magnetic Multilayers

A multilayer with stacking order of Ta(2)/Ir(3)/Fe_0.65_Tb_0.35_(6)/Ru(1.1)/Co(1)/Pt(3)/Ta(2) (thickness in nanometers) is deposited on oxidized silicon substrates utilizing ultrahigh vacuum magnetron sputtering system, in which the compensated Fe_0.65_Tb_0.35_ ferrimagnetic layers are synthesized through co‐sputtering Fe and Tb targets. As shown in Figure 1a, the designed SAF magnetic multilayers structure primarily is consisted of a ferromagnetic Co layer, an intermediate Ru layer, and an ferrimagnetic FeTb layer. Notably, the magnetic property of Fe_0.65_Tb_0.35_ ferrimagnet is Tb‐dominant, where the net magnetization direction ($\overset{\to }{\underset{\mathrm{Fe}-\mathrm{Tb}}{M}}$) is parallel to the magnetization direction ($\overset{\to }{\underset{\mathrm{Tb}}{M}}$) of Tb sublattice, while the magnetization direction of Fe sublattice ($\overset{\to }{\underset{\mathrm{Fe}}{M}}$) is antiparallel to the $\overset{\to }{\underset{\mathrm{Tb}}{M}}$ [29, 30]. The Ru layer, as a nonmagnetic ultrathin metallic layer, mediates the interlayer exchange coupling in the form of an interlayer RKKY interaction between FeTb layer and Co layer [39, 40]. To validate the interfacial roughness and element distribution for the SOT performance of SAF device, the high‐angle annular dark‐field scanning transmission electron microscope (HAADF STEM) and the corresponding energy dispersive spectrometer (EDS) are measured. The experimental results indicate that the multilayer structures exhibit smooth and flat interfaces accompanied by distinct elemental distribution as shown in Figure 1b. EDS elemental mapping reveals a highly uniform distribution of Fe and Tb element. The write current is appiled along longitudinal *x*‐axis direction (the direction of in‐plane magnetic field) while AHE voltage is measured from the Hall voltage along the *y*‐axis direction. The apparent overlap and somewhat diffuse contrast in the elemental mappings are attributed to the intrinsic limitations of the EDS technique, where the interaction volume of the electron beam exceeds the atomic‐scale periodicity of the material. The HAADF‐STEM image, possessing higher spatial resolution, confirms the uniform amorphous microstructure of the film, which is consistent with the EDS results indicating a homogenous composition at the nanoscale. The magnetic and electrical transport measurements are examined by utlizing a VSM, AHE and a home‐built electrical transport measurement system (Figure 1c). The experimental results indicate the presence of excellent PMA in the SAF structure, as shown in Figure 1d. The ferromagnetic Co layer and ferrimagnetic FeTb layer have significant antiferromagnetic interlayer exchange coupling intermediated by Ru layer and the net magnetization (|$\overset{\to }{\underset{\mathrm{net}}{M}}|$ = $|{\vec{M}}_{\mathrm{Fe}-\mathrm{Tb}}|$ − $|{\vec{M}}_{\mathrm{Co}}|$) of the SAF can approach nearly zero at *H*_z_ = 0 Oe.

**FIGURE 1:**
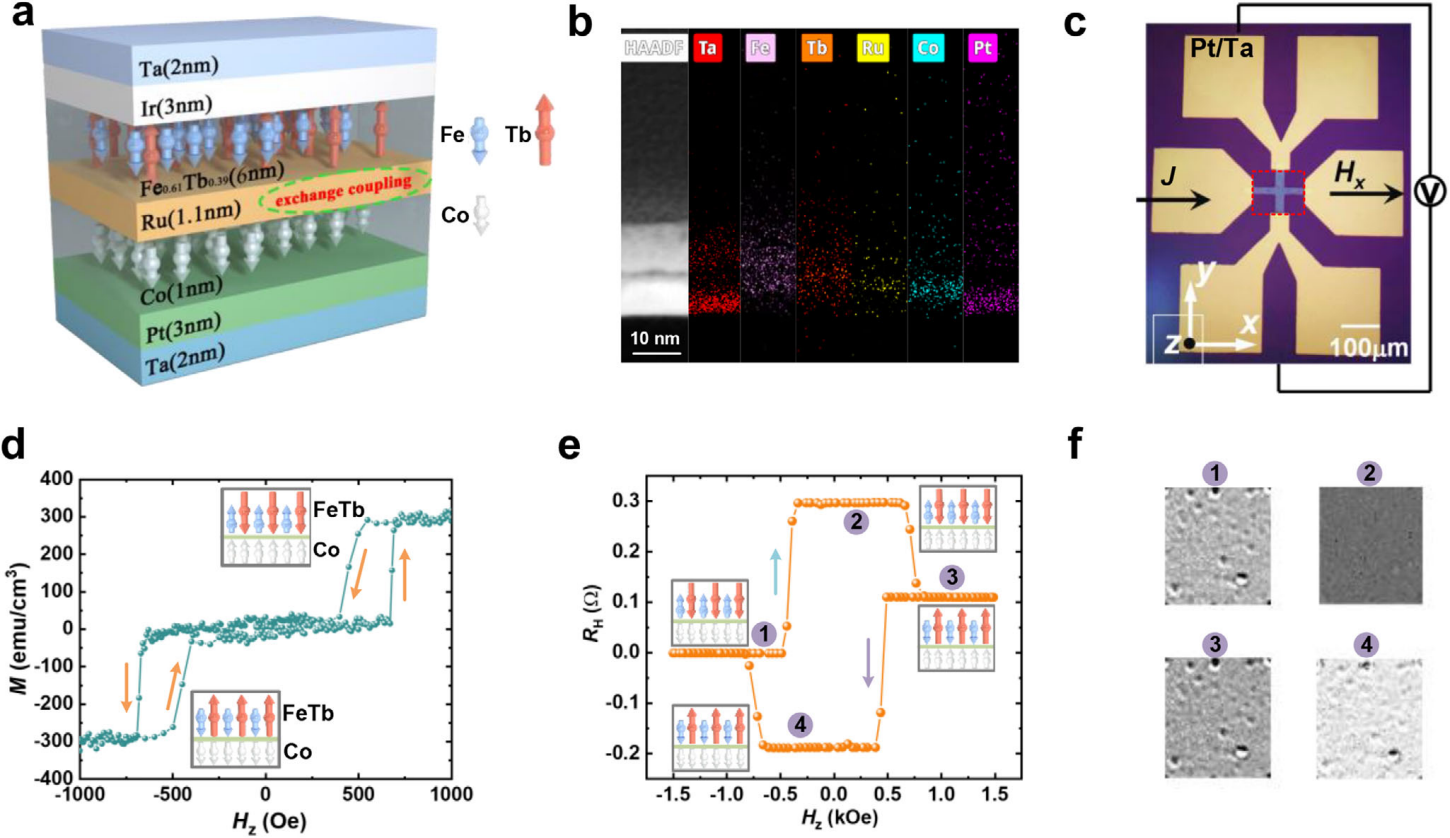
Device structure of the magnetic multilayer SAF and corresponding magnet‐electric transport measurements. (a) Schematic illustration of Ta(2)/Ir(3)/FeTb(6)/Ru(1.1)/Co(1)/Pt(3)/Ta(2) SAF multilayer. (b) HAADF image and EDS elemental mapping of the multilayer SAF structure. (c) An optical image of the Hall bar device and the corresponding measurement configuration. (d) The magnetic hysteresis loops of the multilayer. (e) The corresponding AHE loop. The insets depict four different arrangements of magnetic moments ($\overset{\to }{\underset{\mathrm{Fe}-\mathrm{Tb}}{M}}$↑ $\overset{\to }{\underset{\mathrm{Co}}{M}}↑$, $\overset{\to }{\underset{\mathrm{Fe}-\mathrm{Tb}}{M}}$↑ $\overset{\to }{\underset{\mathrm{Co}}{M}}↓$, $\overset{\to }{\underset{\mathrm{Fe}-\mathrm{Tb}}{M}}↓$ $\overset{\to }{\underset{\mathrm{Co}}{M}}↑$, $\overset{\to }{\underset{\mathrm{Fe}-\mathrm{Tb}}{M}}↓$ $\overset{\to }{\underset{\mathrm{Co}}{M}}↓$). The silver‐white arrow, blue arrow, and red arrow represent the magnetic moments of Co, Tb, and Fe, respectively. (f) The dynamic process of magnetization reversal is captured by the p‐MOKE imaging microscope at the selected magnetic fields.

Then the magnetic multilayer film is patterned into Hall bar device through standardized photolithography and Ar‐ion beam etching, while the metal electrodes Ta/Pt are sequentially deposited onto the materials as schematically shown in Figure 1c. The shape of the hysteresis and AHE loops can be understood as follows: the magnetization of the Co layer ($\overset{\to }{\underset{\mathrm{Co}}{M}}$) first reverses its direction at −500 Oe (before zero field) and then the net magnetization of the FeTb layer ($\overset{\to }{\underset{\mathrm{Fe}-\mathrm{Tb}}{M}}$) reverses its orientation at +750 Oe, through sweeping *H*_z_ from −1500 to +1500 Oe. In the full loop, four different magnetization configurations can be identified, which are schematically illustrated in the inset of Figure 1e. The polar magneto‐optical Kerr effect (p‐MOKE) images in selected magnetic fields are recorded in Figure 1f, in which dark and bright colors correspond to parallel and antiparallel states of $\overset{\to }{\underset{\mathrm{Fe}-\mathrm{Tb}}{M}}$ and $\overset{\to }{\underset{\mathrm{Co}}{M}}$, respectively.

### 2.2 Field‐Free Magnetization Switching by Current‐Induced SOT in SAF Device

To study the current‐induced magnetization switching in SAF device, AHE resistances (*R*_H_) are measured by sweeping the the pulse current bidirectionally (−100 mA to +100 mA) under various in‐plane magnetic fields (*H*_x_) varying from −2200 Oe to +2200 Oe. As indicated in Figure 2a, the polarity of magnetization switching exhibits clockwise (anticlockwise) under positive (negative) *H*_x_ [41]. Generally, magnetization switching of perpendicular magnetic moments driven by SOT requires applying the external magnetic field along the *x*‐direction to break the symmetry. However, it is noteworthy that this magnetization switching can still be achieved when the applied magnetic field is removed in the present SAF devcies. This typical field‐free magnetization switching holds significant promise for development of low‐power and electromagnetic interference‐resistant device due to the lack of an auxiliary magnetic field [42]. To intuitively investigate the magnetization switching efficiency, Δ*R*_H_ is extracted and displayed in Figure 2b. The SOT‐induced magnetization switching ratio is calculated to be ≈50% by Δ*R*_H_/*R*_H_ under zero external magnetic field, where Δ*R*_H_ are the change of AHE resistance induced by the current. The field‐free switching capability originates from the intrinsic symmetry breaking inherent to the SAF structure. In our SAF systems, the combination of strong antiferromagnetic interlayer exchange coupling (mediated by the Ru spacer) and the chiral interlayer Dzyaloshinskii–Moriya interaction (DMI) generates an effective internal bias field that lifts the degeneracy of the two switching directions, enabling deterministic spin‑orbit torque switching without the need for an external magnetic field. A detailed description of the microscopic switching dynamics, including the sequential reversal of the Co and FeTb layers and the role of interlayer DMI in stabilizing chiral domain walls, is provided in the Supporting Information (Figure S1). The Ta heavy metal layer serves as a buffer layer to optimize the PMA growth of FeTb [41, 42]. Effective control of the AHE resistances can be achieved by adjusting the amplitudes and width of the current pulses. Shown in Figure 2c is the SOT‐induced magnetization switching loops of AHE resistances as a function of the pulse current with the duration being fixed at 0.1 ms, which can be obtained by sweeping the writing pulse current ranging from +80 mA to a progressively smaller negative current *I*_x_ (*I*_x_ = ‐40, ‐41, ‐42, and ‐80 mA) and then reverse back to +80 mA. As the magnitude of the negative current *I*_x_ exceeds that of the critical switching current (‐35 mA), the magnetization gradually reverses from its initial state and reaches saturation when the current is up to ‐45 mA. The critical switching current density ***J***_c_ is calculated to be approximately ‐1.1 × 10^7^ A/cm^2^ with width W = 20 µm and the total thickness of the magnetic films *t* = 18.1 nm. This dynamic process reflects the gradual nucleation switching process induced by SOT. In addition, the curves exhibit multiple non‐volatile stable AHE resistances upon the elevation of current pulse amplitude due to the existence of multidomain states in SAF. The typical memristive behavior, which remains even after the applied current is removed, plays a crucial role in mimicking the functionalities of artificial synapses and neurons. And then the SOT‐induced magnetization switching loops by feeding various widths of pulses (pulse width = 0.1 , 0.5, 1, 5, and 10 ms) are also systematically studied. Figure 2d reveals the dynamic process of magnetic moment reversal, namely the reversed domain nucleation and propagation. This evolutionary process—from a uniform dark state to the appearance of bright spots at the device edges, followed by the gradual expansion of these spots until they eventually merge into a uniform bright state—is in agreement with reported work of magnetic domain nucleation and propagation under current‐driven SOT conditions [43]. These bright spots correspond to localized reverse magnetic domains, which preferentially nucleate at the device edges due to the higher demagnetizing field strength there. Under the influence of a sustained current pulse, the subsequent growth of these bright spots reflects the depinning and propagation of chiral domain walls, which is precisely the microscopic origin of the gradual resistance change observed in Figure 2d. Seen in Figure 2e, the critical switching current gradually decreases while the change of AHE resistances increase with the increasing of pulse width, indicating that higher switching efficiency can be achieved with a smaller current. This phenomenon is particularly pronounced when applying current pulses with pulse width exceeding 1 ms, which may be attributed to the effects of current‐induced Joule heating [44, 45]. The temporal stability and cyclic stability of SAF device are further verified (seen in Figure S2). In summary, the studtied SAF device exhibits field‐free magnetization switching and multiple non‐volatile stable states by altering current amplitude/width to realize memristive behavior for neuromorphic computing. Therefore, achieving field‐free SOT switching not only simplifies device design by eliminating the magnetic field generation component but also reduce energy consumption and enhance the robustness of devices in practical applications.

**FIGURE 2:**
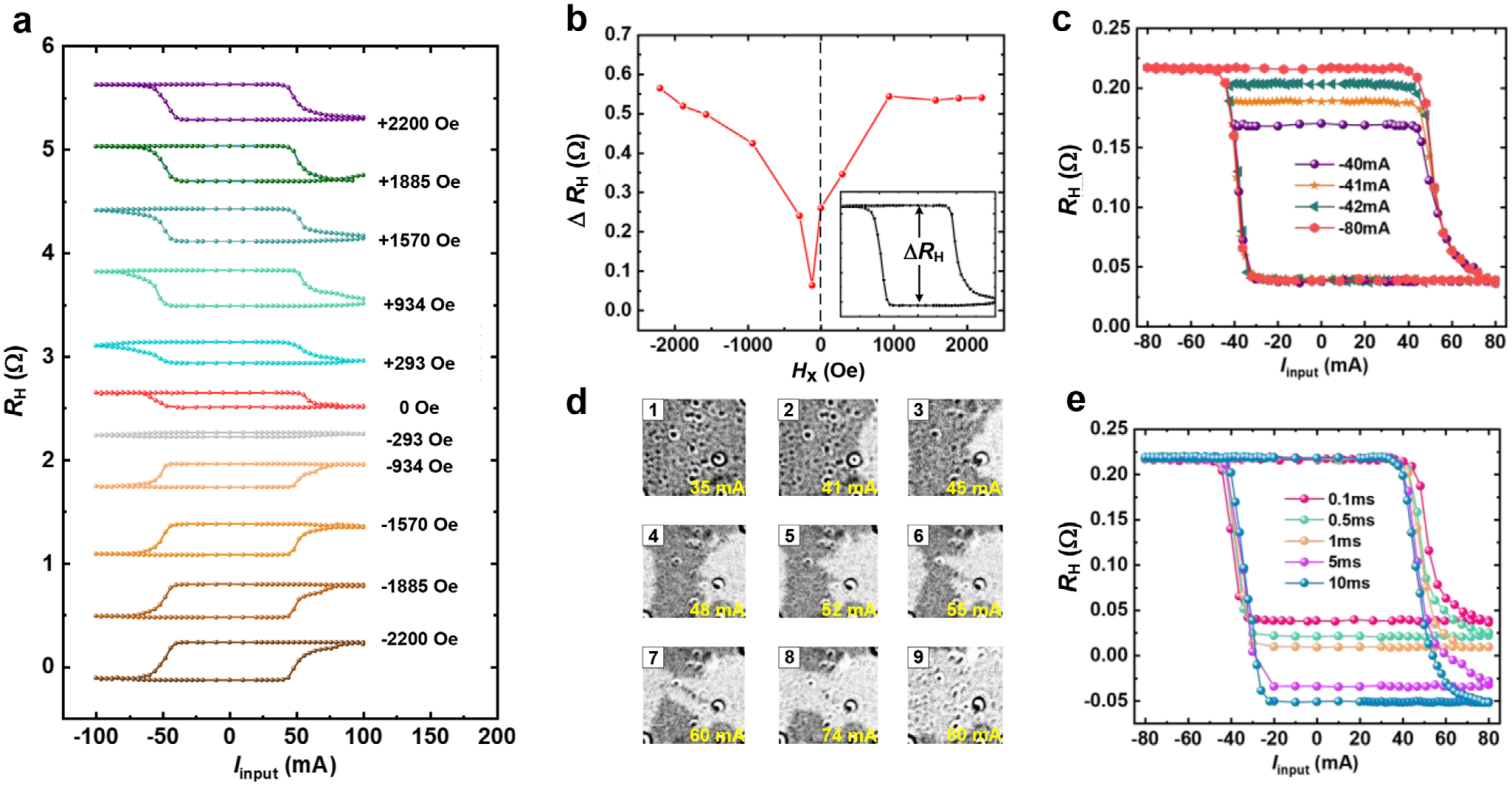
SOT‐induced magnetization switching in SAF device. (a) AHE loops under various in‐plane magnetic fields *H*_x_ ranging from −2200 Oe to +2200 Oe (bottom to top, respectively). The red curve denotes the switching loop under *H*_x_ = 0 Oe. (b) The change of AHE resistances as a function of *H*_x_. (c) The curves of AHE resistance *R*_x_ variations under zero magnetic field as the current pulse amplitude increases. The current is swept from +80 mA to a progressively smaller negative current *I*_x_ (*I*_x_ = −40, −41, −42, and −80 mA) and then reverses back to +80 mA. (d) The p‐MOKE images corresponding to magnetic domain nucleation and propagation. (e) The current‐induced switching curves were measured by applying the current pulse of different widths.

### 2.3 Radiation Resilient SAF Devices

Excellent radiation tolerance is another crucial property for evaluating neuromorphic hardware under extreme conditions. Benefiting from the inherent zero net magnetic moment and strong antiferromagnetic exchange coupling effects, SAF can maintain magnetic order stability more effectively than traditional ferromagnetic materials, exhibiting greater resistance to failure caused by radiation‐induced magnetic moment perturbations [46]. The potential of SAF to serve as radiation‐resistant, highly stable spintronic devices has been widely recognized among researchers, although few studies have directly reported their radiation resistance. In this section, we primarily explore the magnetic moment switching performance of SAF devices by varying total irradiation doses (TIDs), providing a novel approach for future neuromorphic applications in extreme environments.

The SAF devices are irradiated at a rate of 150 krad/h utilizing a Co‐60 γ‐ray radiation source to simulate a realistic γ radiation environment, which can be modulated by adjusting the distance of the sample from the γ‐ray source. Notably, vacuum pre‐treatment is required prior to irradiation to ensure the removal of ambient moisture and oxygen. The SOT‐induced magnetization switching curves with an increasing TIDs of up to 1 Mrad are shown in Figure 3a. Negligible effect on the magnetic properties of the SAF devices is observed upon radiation, indicating that the devices exhibit excellent radiation resistance. To obtain microscopic insight into the superior radiation tolerance of the SAF structure, the micromagnetic simulations comparing the magnetization stability of a single Co ferromagnetic layer and the FeTb/Ru/Co SAF under random defect distributions (mimicking radiation‑induced damage) are performed. The simulation results reveal that the SAF under defects is significantly less affected by disturbances during the switching compared to the single‐layer FM, which indicates that the SAF interlayer coupling could effectively suppresse defect‑induced perturbations. A detailed description of the simulation setup and results is provided in Figure S3. Although SAF devices exhibit great radiation resistance under high‐dose irradiation, both the free‐layer interface and heavy‐metal layer of the thin‐film structure sustain irreversible damage at higher radiation doses [39]. To quantitatively characterize the impact of TIDs on SAF devices, we extract the difference (Δ*R*_H_) between the high‐resistance state (HRS) and low‐resistance state (LRS), as well as the threshold reversal current (*I*_th_) as evaluation metrics. As shown in Figure 3b, Δ*R*_H_ and *I*_th_ decreases slowly with increasing TIDs, which is possibly attributed to radiation‐induced defects [47]. These defects generated by radiation in the Co layer, the FeTb ferrimagnetic layer, and their interface act as pinning centers for magnetic domains. The movement of domain walls requires additional energy to overcome these pinning points, necessitating a stronger external field (the effective field generated by the current) to drive magnetic moment reversal [48]. Table S1 presents the statistical results for Δ*R*_H_ and *I*_th_ at different TIDs extracted from Figure 3b, which indicates that the devices retain over 92% performance after 1 Mrad (Si) γ‐irradiation. Therefore, the fabricated SAF devices work well even after exposure to a TID of up to 1 Mrad, which is sufficient to handle the operating environments (lower than 500 krad generally) of the vast majority of current devices

**FIGURE 3:**
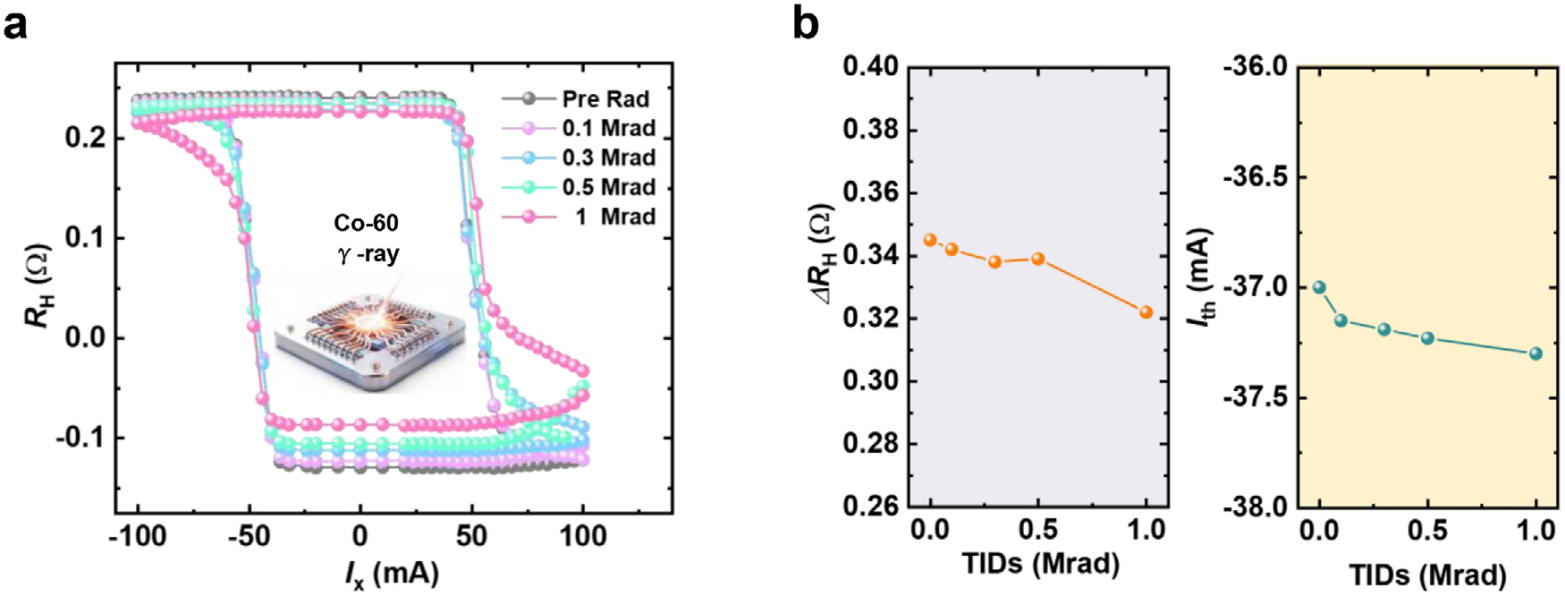
Radiation effect measurements of SAF‐based devices. (a) The magnetization switching of the SAF devices before and after irradiation under different TIDs (0, 0.1, 0.3, 0.5, and 1 Mrad). The inset simulates the device receiving γ radiation from all directions with Co‐60 serving as the radiation source. (b) The difference between the initial state and the saturated state (left panel), the threshold reversal current before and after irradiation (right panel) under different TIDs.

### 2.4 SAF‐Based Artificial Synapses and Neurons

Based on the pulse‐regulated field‐free magnetization switching responses in SAF devices, the behavior of artificial synapses and neurons is successfully mimicked. Figure 4a shows a schematic diagram of a FeTb‐based SAF artificial synapse artificial synapses and neurons. The AHE resistances measured during the magnetization switching is defined as the weight of the artificial synapse or as the nervous impulse of the artificial neuron.

**FIGURE 4:**
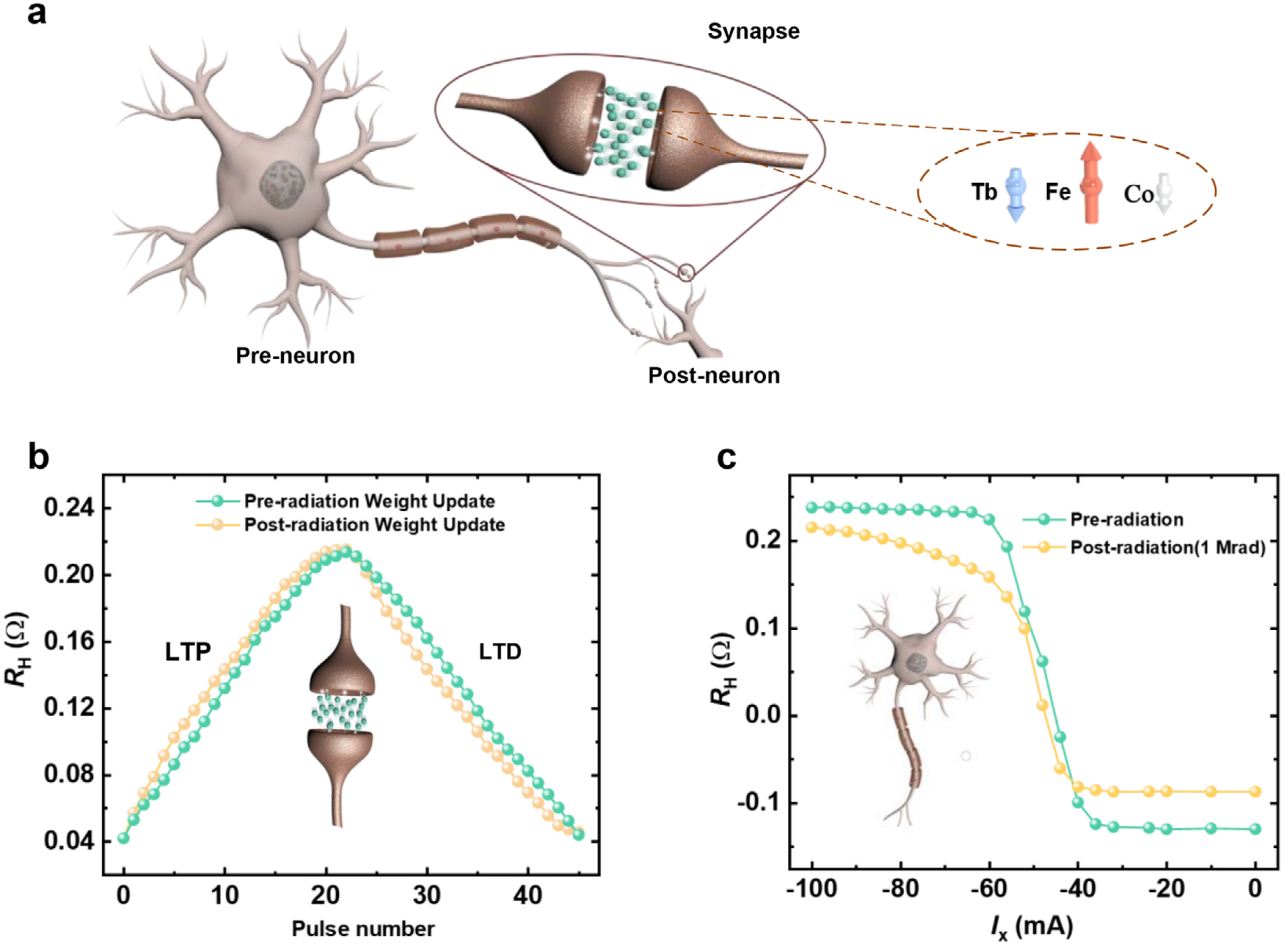
SAF‐based artificial synapse and neuron. (a) Schematic illustration of neuron and synapse structure. (b) The demonstration of LTP and LTD that is enabled by applying potentiation (depression) training pulse with a pulse width of 1 µs before and after radiation. (c) The observed non‐linear curve of AHE resistance as a function of the current pulse *I*_x_ before and after radiation.

The reversal of the ferromagnetic layer and ferrimagnetic layer magnetizations causes a change on AHE resistances, which can be utilized to characterize the change of the synaptic weight. The current‐induced switching between two synthetic antiferromagnetic states can effectively emulate the long‐term depression (LTD) and long‐term potentiation (LTP) of the synaptic weight. The linear LTP and LTD processes of the SAF synaptic weight can be regulated by applying appropriate pulse currents amplitudes/widths. We first apply 22 consecutive negative pulses with a fixed pulse width of 1 µs and increase current between 45 and 55 mA, realizing the behavior of LTP. Subsequently, the behavior of LTD is emulated by applying 22 consecutive positive pulses. The variation of AHE resistances modulated by the SOT‐induced magnetization switching, can be used to represent the synaptic weight. By setting current pulses in the range between 45 and 55 mA, the SAF artificial synapse is able to imitate the linear variations of synaptic weights during the LTP and LTD processes (Figure 4b).

In addition to the required artificial synapses, neuromorphic computing also demands that artificial neurons provide non‐linear activations for weighted signals. In the biological nervous system, neurons integrates the output signals and transmitting them to the next layer of neurons through interconnected synapses. For the state‐of‐art artificial neuronal operations, the received weighted signals need to be activated non‐linearly by the artificial neuron. Thus, the nonlinear behavior of pulse‐induced magnetization switching of SAF measured previously holds significant value for simulating artificial neurons. Figure 4c exhibits the evolution of AHE resistance as a function of current intensities (with a fixed pulse width of 10 µs) before and after radiation. The “S‐shape” AHE resistance curve obtained by scanning current pulses from −100 to 0 mA bears similarities to the SIGMOD activation function—a well‐known function widely used in neural networks. By comparison, we observe that the reverse slope of the activation function curve becomes less steep after radiation, yet the overall shape remains S‐shaped. The nonlinear AHE resistances can be explained by the non‐uniform magnetization reversal process (namely, the reversed domain nucleation and propagation processes). It is worth noting that a reset operation need to be performed before it can function as a neuron due to the non‐volatility of the SOT‐switching in SAF. In summary, we have successfully simulated artificial neurons and synapses by employing the field‐free magnetization switching in SAF and will explore its applications in neuromorphic computing in the following sections.

### 2.5 SAF‐Based Neuromorphic Device for SST Reconstruction

Subsequently, we deeply integrate the observed physical characteristics of SAF with the cross‐attention mechanism of the Perceiver IO architecture to perform SST reconstruction tasks. Compared to the previously widely reported image recognition tasks based on synaptic devices, this nonlinear spatiotemporal physical field reconstruction task is more complex [49]. Furthermore, devices deployed for long‐term operation require to contend with complex and dynamic electromagnetic environments. The current mainstream Perceiver IO neural network model effectively addresses the mapping problem from discrete sensor readings to continuous temperature fields by learning latent arrays and cross‐attention mechanism. We find that the experimentally observed nonlinear AHE resistance changes share mathematical properties and information processing requirements similar to those of attention curves: the threshold voltage of the SAF corresponds to the critical point where the attention mechanism focuses, while conductance saturation corresponds to the physical implementation of weight normalization. At the same time, the aforementioned SAF devices demonstrate excellent radiation resistance under high‐dose radiation exposure, providing a strong guarantee for edge device deployment. Therefore, integrating the advantages of SAF and beyond, we propose a neuromorphic computing framework based on SAF devices for reconstructing ocean surface temperature fields.

As shown in Figure 5a, the simulation process can be divided into the following sections including input encoding, SAF cross‐array computation, implementation of the physical attention mechanism, forward network refinement and output decoding and temperature field reconstruction. The model inputs (*X*) include the latitude, longitude, and temperature values of a sensor at a given moment (seen in Equation (S1) of Supporting Information S4). Subsequently, The features are mapped to the operating range of the SAF device through normalization by feature‐level fusion strategy (seen in the Equations (S2–S5) of Supporting Information S4). The advantage of this encoding strategy lies in fusing position information and temperature observations at the feature level, rather than simply concatenating them. Position encoding captures spatial relationships between sensors via the tanh function, while temperature encoding applies nonlinear transformations to observations using a modified sigmoid function fitted based on SAF experimental results (seen in Figure 5c). Ultimately, deep feature interaction is achieved through element‐wise multiplication of them (seen in the Equation (S6) of Supporting Information S4). Traditional Perceiver IO‐based cross‐attention mechanisms compute attention scores by calculating the similarity between *Q* and *K*, then normalize them into attention weights, and finally perform weighted summation over *V* [50]. The nonlinear conductance characteristics in studied SAF devices can simulate the competitiveness within the attention mechanism and the cross‐array structure naturally implements weighted summation and normalization based on Kirchhoff's law. Specifically, the similarity *S* between the *Q* and *K* is used as the programming signal for SAF devices and adjusting its conductance values. And then the vector *V* is applied as the input current to the cross array, and the output current is the attention output O we require. The *V* is then applied as the input voltage to the crossbar array and the output current represents the desired attention output *O*. The unique physical attention mechanism implemented by SAF offers distinct advantages over digital softmax. First, the nonlinear response of SAF devices exhibits a mathematical form highly similar to softmax, both displaying S‐shaped curve characteristics. Second, the natural saturation properties of conductance automatically realize the physical mechanism of weight competition, eliminating the need for explicit exponential and division operations. Furthermore, the attention output *O* is refined through two layers of feedforward networks (i.e., the MLP connected after the attention mechanism, as interpreted in Equations ((1), (2), (3)), where *f_SAF_* is the modified activation function shown in Figure 5c.

(1) $$H_{1}=f_{\mathrm{SAF}}\left(G_{\mathrm{fc}1}\cdot O+I_{\mathrm{bias}1}\right)\in R^{N\times M}$$

(2) $$H_{2}=f_{\mathrm{SAF}}\left(G_{\mathrm{fc}2}\cdot H_{1}+I_{\mathrm{bias}2}\right)\in R^{N\times M}$$

(3) $$H_{\mathrm{final}}=H_{2}+O\in R^{N\times M}$$

**FIGURE 5:**
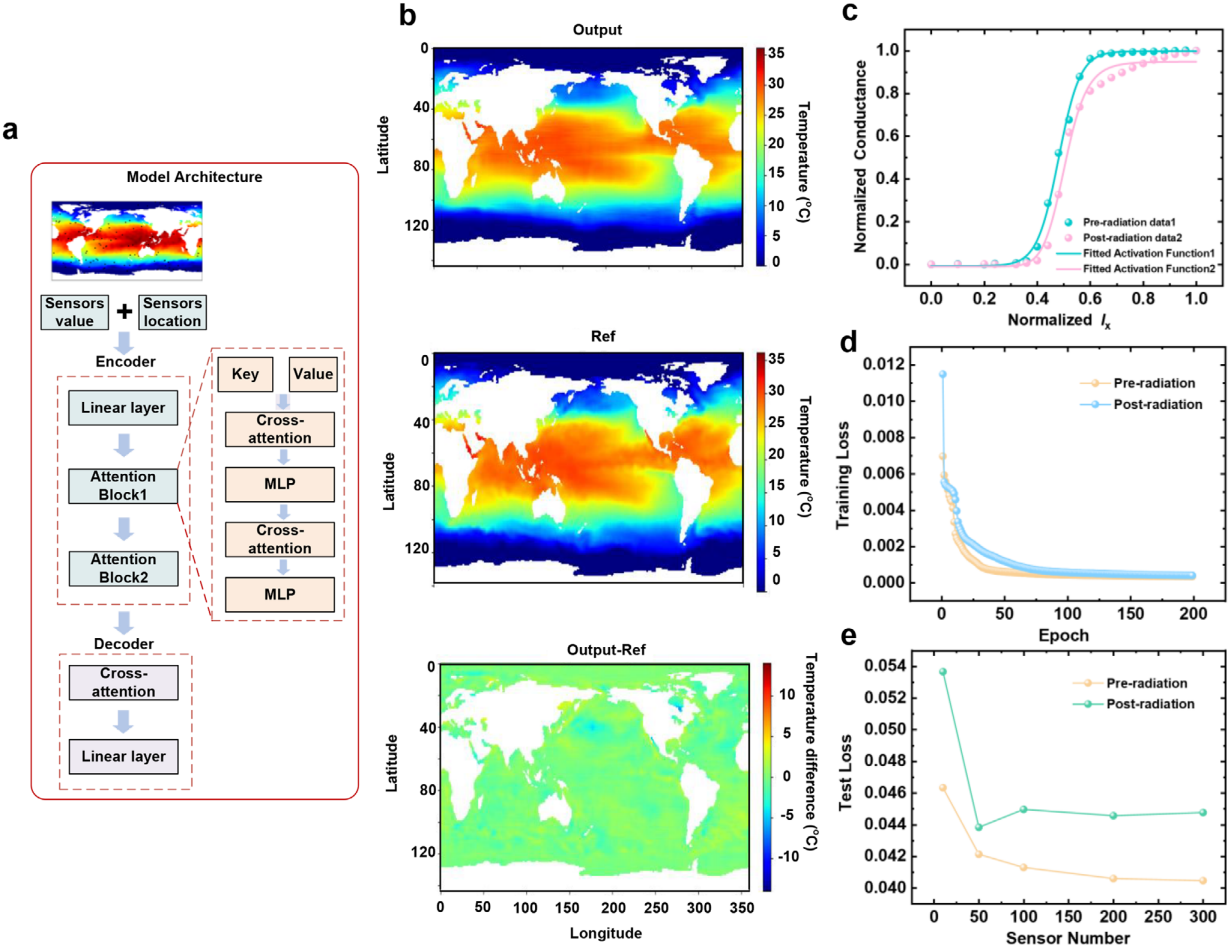
Neuromorphic simulation based on the nonlinear physical characteristics of SAF. (a) Overview of SST reconstruction using the Perceiver IO neural network model. (b) The actual output temperature value, the reference value, and the difference between the two corresponding to the top, middle, and bottom inset, respectively. (c) The nonlinear activation function of the artificial neurons before and after radiation. (d) Training loss as a function of epoch. (e) Test loss as a function of sensor number.

The feedforward network ensures nonlinear transformation and feature refinement of the attention outputs *O*, where *G*_fc1,2_ represents the conductance matrix of the feedforward network. Residual connections *H*_final_ guarantee the stability of ground‐level flow while preserving the original attention information. Finally, the actual temperature *T*_final_ is obtained by mapping the fixed‐length latent representation to a temperature field of arbitrary dimensions and performing denormalization.

Note that the SST data used in the simulation calculations is sourced from the ERA5 reanalysis dataset provided by the National Oceanic and Atmospheric Administration (NOAA). Figure 5b shows the reconstruction results from the model integrating the nonlinear physical characteristics of SAF. The results indicate that the reconstruction quality is satisfactory, with a clear overall temperature field structure and errors within 2°C. Compared to mainstream deep learning methods, the SST reconstruction achieved by integrating the nonlinear response of our SAF devices with a Perceiver IO architecture is projected to consume tens of times less energy. Figure 5c is the fitted activation function of the artificial neurons before and after radiation. The modified SIGMOD function is obtained by fitting the characteristics of the experimental data (The detailed fitting process is shown in the Equation (S18) of Supporting Information S5). Figure 5d,e shows the variation in loss values under different training iterations and numbers of tested sensors, respectively. Although mainstream deep learning algorithms currently achieve reconstruction errors below 1°C in marine reconstruction tasks, the neuromorphic algorithm proposed in this study—based on the integration of SAF and attention mechanisms—meets application requirements in terms of reconstruction accuracy. It also demonstrates significant advantages in energy efficiency, reliability, and environmental adaptability, offering a novel technical pathway for intelligent computing in marine edge computing. Future work will focus on the integrated fabrication of SAF cross arrays and further optimization of the physical‐algorithmic co‐design, driving the practical application of neuromorphic computing in fields such as ocean monitoring.

## 3 Conclusions

In summary, we fabricate a Pt/Co/Ru/FeTb/Ir SAF neuromorphic devices with field‐free magnetization switching, which not only enables continuous modulation of AHE resistance to simulate biological synapses and neurons, but also exhibit excellent radiation resistance (below 8% performance degradation under 1 Mrad radiation exposure). Benefiting from the aforementioned advantages, we achieve a neuromorphic computing framework based on SAF synaptic devices for robust SST reconstruction through physical‐algorithmic co‐design. The simulated SST reconstruction results could perform <2°C RMS error comparable to mainstream deep learning (<1°C RMS error) and has the potential to reduce energy consumption by tens of times compared to optimized digital integrated circuits running on neural network models of equivalent depth [51, 52]. This work provides a novel device‐algorithm co‐optimization pathway for low‐power, high‐reliability edge intelligent computing in extreme environments, holding significant implications for advancing the deployment of neuromorphic computing in practical scenarios such as marine monitoring and climate prediction. Future efforts will focus on advancing the integrated fabrication of SAF crossbar arrays, conducting long‐term deployment tests in real marine environments, and further deepening the synergistic optimization of physical properties and algorithmic requirements.

## 4 Experimental Section

### 4.1 Experiment Details

Magnetic multilayer of stacking Ta(2)/Ir(3)/Fe_0.65_Tb_0.35_(6)/Ru(1.1)/Co(1)/Pt(3)/Ta(2) (the numbers denote the thickness in nanometer) was fabricated on thermally oxidized silicon substrates using magnetron sputtering system (AJA Orion 8). The base pressure of the main chamber was better than 1 × 10^−8^ Torr and the Ar pressure was at 3.0 mTorr. A 2 nm thick Ta was used as adhesive layer for preventing the oxidization of the underlying FeTb layer. respectively. The FeTb layer was synthesized through co‐sputtering of the Fe and Tb targets. The multilayer film was patterned into a crossbar device with a channel width of 10 µm by using standard photolithography and ion beam etching. The magnetometry and electrical transport property measurements were performed by utilizing VSM and a home‐built electrical transport measurement system. The magnetization configuration images were taken by a polar MOKE imaging microscope from evico magnetics (Dresden).

### 4.2 SST Reconstruction Simulations

The sea surface temperature ERA5 reanalysis data provided by the National Oceanic and Atmospheric Administration (NOAA) used in this paper has a temporal resolution of 1 week and a spatial resolution of 1°, forming a 360 × 180 matrix. During the training phase, we utilized 1040 matrices spanning the period from 1981 to 2001. For the testing phase, data from 2001 to 2018 was employed. The number of sampling points was set to 10, 50, 100, 200, and 300, with L2 loss serving as the evaluation metric. Input data entering the model consists of sensor values concatenated with position values.

## Conflicts of Interest

The authors declare no conflicts of interest.

## Supporting information




**Supporting File**: advs76388‐sup‐0001‐SuppMat.docx.

## Data Availability

The data that support the findings of this study are available from the corresponding author upon reasonable request.
