# Supplementary material for: Radiation Resilient Synthetic Antiferromagnets‐Based Neuromorphic Device for Sea Surface Temperature Reconstruction
Source: Adv Sci (Weinh). 2026 Jun 27:e76388. Online ahead of print. doi: 10.1002/advs.76388 (PMC13336405; doi:10.1002/advs.76388)
Supplement: Supplementary file 1 — Supporting File: advs76388‐sup‐0001‐SuppMat.docx. [file ADVS-9999-e76388-s001.docx]

**Supporting Information**

**Radiation Resilient Synthetic Antiferromagnets-Based Neuromorphic Device for Sea Surface Temperature Reconstruction**

Mingxu Song ^1,4,⊥^, Jiahao Liu^1,4,⊥,^*, Ruisheng Hu^2^, Teng Xu^3^ , Aihua Tang^3,^*, and Zhihong Zhu^1,4,^*

*1 College of Advanced Interdisciplinary Studies,* *National University of Defense Technology, Changsha, China, 410073.*

*2 College of Meteorology and Oceanography, National University of Defense Technology, Changsha, China, 410073.*

*3 Anhui Province Key Laboratory of Low-Energy Quantum Materials and Devices, High Magnetic Field Laboratory, Hefei Institutes of Physical Science, Chinese Academy of Sciences, Hefei, China, 230031.*

*4 Nanhu Laser Laboratory, National University of Defense Technology, Changsha, China, 410073.*

Correspondence to whom should be addressed to: liujiahao20@nudt.edu.cn (Jiahao Liu), [ahtang@hmfl.ac.cn](mailto:ahtang@hmfl.ac.cn) (Aihua Tang) and and zhuzhihong@163.com (Zhihong Zhu)

**S1. Microscopic mechanism of field‑free SOT switching in SAF devices**

The deterministic field‑free switching observed in the FeTb/Ru/Co synthetic antiferromagnetic (SAF) structure can be understood through the symmetry breaking inducrd by the interlayer DMI. The key steps are illustrated schematically in Figure S1 and summarized as follows. In the absence of an external field, the two magnetic layers are antiferromagnetically coupled through the Ru spacer, resulting in antiparallel alignment of their net magnetic moments. The interlayer DMI introduces an antisymmetric exchange coupling between the two magnetic layers, which effectively breaks the in‑plane mirror symmetry of the SAF structure. This symmetry breaking can lift the degeneracy of the two switching directions, enabling deterministic SOT switching without an external magnetic field.^[1, 2]^ Moreover, the chiral nature of the DMI stabilizes the Néel‑type domain walls and determines the polarity of the switching, which is consistent with the observed asymmetry in the critical switching current.^[3]^

**Figure S1.** The microscopic mechanism of field‑free SOT switching in SAF devices

When a current pulse is applied along the +x direction, the spin‑orbit torque (SOT) generated by the Pt heavy metal layer acts primarily on the adjacent Co layer. The partial magnetization of Co layer begins to process coherently and flips to the +z direction. Due to the interlayer antiferromagnetic coupling, a reversed domain simutaneously nucleates in the FeTb layer and there exists a domain wall. The chirality of this domain wall is determined by the intralayer and interlayer DMI througout SAF structure,^[3]^ which in turn defines the preferred direction of domain wall propagation under exchange coupling torque. The stronger intralayer DMI preferly stabilizes the chiral Néel-type wall and breaks the symmetry of the wall motion, allowing the domain wall to be propagated without an external in-plane magnetic field. The reversed domain further expands, which progressively increases the volume fraction of the reversed doamin in both Co and FeTb layer. Lastly, the two layers are again antiparallel, restoring the energetically favored SAF state.

**S2. The operational and temporal stability measurements**

**Figure S2** a) The operational stability measurements of the devices under 1 Mrad irradiation by enabling the “Program” and “Reset” procedures. The measurement process is repeated up to 100 times by applying a positive/negative saturated current pulse at room temperature. b) The temporal stability measurements by comparing AHE loops taken one year apart.

We futher test the operational stability of the SAF devices after radiation by repeatedly executing the “Program” and “Reset” procedures as shown in Figure S2a, which are crucial for its application in neuromorphic computing. The full process is repeated up to 100 times by applying a negative/positive pulse to achieve highest/lowest stable resistance states. Additionally, we conduct the temporal stability tests by comparing AHE loops taken one year apart, as shown in Figure S2b. These tests demonstrate the operational and temporal stability of SAF devices.

**S3. The magnetization stability of SAF structure under the influence of random defects**

We employed micromagnetic simulations (Mumax^3^ platform) to model the electrically driven magnetic switching processes in SAF (FeTb and Co) devices and FM devices (Co), comparing switching dynamics with and without random defects. In the micromagnetic simulation: The grid size was set to 4 nm × 4 nm × 1 nm, and the device geometry is defined as 128 nm × 128 nm, accommodating the formation and evolution of multi-domain structures. Material parameters were set as follows: Perpendicular magnetic anisotropy energy ***K***_u_ = 310 J/m^3^ (Co), ***K***_u_ = 290 kJ/m^3^ (FeTb), Saturation magnetization ***M***_s_ = 640 kA/m (Co), ***M***_s_ = 600 kA/m (FeTb), exchange constant *A* = 8 × 10⁻^12^ J/m, and damping α = 0.02. For external excitation, a current density ***J*** = 2.1×10^7^ A/cm^2^ was applied with pulse width of 5 ns and separated by 10 ns intervals. Radiation effects were simulated by introducing random defects at 10% ***K***_u_ during the simulation. Due to the non-volatility of magnetic materials, neither the SAF nor the FM exhibited significant defect-induced effects in the stable state. During electrically driven reversal, the SAF exhibited negligible defect influence (Figure 3a). In contrast, the FM demonstrated markedly increased final in-plane magnetization component ***M***_z_ fluctuations throughout the pulse duration, even exhibiting one logic reversal error (Figure S3b). This indicates that the interlayer antiferromagnetic coupling in the SAF provides additional radiation resistance during electrically driven reversal.

**Figure S3**. Simulation of the electrically driven magnetic reversal process in a) SAF and b) FM devices, with a comparison of the reversal process with and without random defects. c) Evolution of the magnetic moment of FM after the introduction of defects.

**S4. SST Reconstruction Simulations process**

The input model can be explained by Formula (1): Here, *P* contains the sensor's latitude and longitude coordinates, while the temperature vector T contains the corresponding temperature values. This position and temperature information are fused via feature-level fusion strategy (Formula (2), (3) and (4)), then normalized and mapped to the operating range of the SAF device (Formula (5)). Noting that $\cdot$ denotes element-wise multiplication, where M is the feature dimension. Immediately following the encoding of the current signal, the query *Q*, key *K*, and value *V* are generated based on SAF cross array. Among these, *G*, L and σ respectively represent the conductance matrix of the SAF obtained through a nonlinear mapping, potential array length and the degree of nonlinearity in the control mapping.

The detailed calculation process can be explained by the following Formula (8), (9), (10) and (11), where $\sqrt{d_{k}}$ denotes the *K* vector dimension, each element *S*_ij_ of *S* represents the matching degree between the i-th *Q* and the j-th *K*, and σ represents the nonlinear activation function based on the physical properties of SAF devices, *A*_norm_ denotes the normalized weight, and *O* denotes the attention output. where H and W represent the target grid dimensions ( Formula (15), (16) and (17)).

$X=[P, T_{sensor}]\in R^{N\times3}$ (1)

$F_{pos}=tanh(W_{pos}\cdot P+b_{pos})\in R^{N\times M}$ (2)

$F_{temp}=\sigma(W_{temp}\cdot T_{sensor}+b_{temp})\in R^{N\times M}$ (3)

$I_{fused}=F_{pos}\cdot F_{temp}\in R^{N\times M}$ (4)

$I_{fused}=I_{min}+(I_{max}-I_{min})\cdot\frac{I_{fused}-min(I_{fused})}{\max\left( I_{fused} \right)-min(I_{fused})}\in R^{N\times M}$ (5)

$Q、K、V=G_{Q、K、V}\cdot I\in R^{L\times M}$ (6)

$G=G_{min}+(G_{max}{-G_{min})\cdot\sigma(\alpha\cdot(W-W_{0}))}$  (7)

$S=Q\cdot K^{T}/\sqrt{d_{k}}\in R^{L\times N}$ (8)

$R=R_{min}+(R_{max}{-R_{min})\cdot\sigma(\beta\cdot(S-S_{0}))}$ (9)

$A=1\cdot R\in R^{L\times N}$ (10)

$O=A_{norm}\cdot V\in R^{L\times N}$ (11)

$T_{flat}=W_{dec}\cdot H_{final}+b_{dec})\in R^{H\times M}$ (15)

$T_{out}={reshape(T_{flat},\left[ H,W \right])}\in R^{H\times M}$ (16)

$T_{final}=T_{min}{+(T_{max}-T_{min})\cdot T}_{out}\in R^{H\times M}$ (17)

**S5. The modified SIGMOD function fitting process**

In the simulated neuron, we use a modified SIGMOD function to fit the characteristics of the experimental data: where *K*, x0, and A represent the slope, the rising point, and the y-proportionality of the modified SIGMOD function with C being the cycle-to-cycle error, respectively.

$y=\frac{A}{1+e^{-\frac{x-x0}{K}}}+C$ (18)

**Table S1.** (Statistical Significance Test for γ-Rays)

| TIDs  [M rad] | Δ*R*_H_  [Ω] | *I*_th_  [mA] | Performance  [%] |
| --- | --- | --- | --- |
| 0 | 0.345 | -37 | 100 |
| 0.1 | 0.342 | -37.1 | 99.1 |
| 0.3 | 0.339 | -37.1 | 98.2 |
| 0.5 | 0.337 | -37.2 | 97.6 |
| 1 | 0.322 | -37.3 | 92.3 |

**References**

[1] W. He, C. Wan, C. Zheng, Y. Wang, X. Wang, T. Ma, Y. Wang, C. Guo, X. Luo, M.E. Stebliy, G. Yu, Y. Liu, A.V. Ognev, A.S. Samardak, X. Han, Field-Free Spin–Orbit Torque Switching Enabled by the Interlayer Dzyaloshinskii–Moriya Interaction, Nano Letters 22(17) (2022) 6857-6865.

[2] Z. Wang, P. Li, M. Fattouhi, Y. Yao, Y.L.W. Van Hees, C.F. Schippers, X. Zhang, R. Lavrijsen, F. Garcia-Sanchez, E. Martinez, A. Fert, W. Zhao, B. Koopmans, Field-free spin-orbit torque switching of synthetic antiferromagnet through interlayer Dzyaloshinskii-Moriya interactions, Cell Reports Physical Science 4(4) (2023).

[3] K. Wang, V. Bheemarasetty, G. Xiao, Spin textures in synthetic antiferromagnets: Challenges, opportunities, and future directions, APL Materials 11(7) (2023).
